# ﻿Species groups, subgroups, and key to world species of the genus *Schizotetranychus* Trägårdh, 1915 (Acari, Prostigmata, Tetranychidae)

**DOI:** 10.3897/zookeys.1211.127353

**Published:** 2024-09-04

**Authors:** Muhammad Kamran, Abdul Hafeez, Fahad Jaber Alatawi, Carlos Holger Wenzel Flechtmann

**Affiliations:** 1 Department of Plant Protection, College of Food and Agriculture Sciences, King Saud University, Riyadh, Saudi Arabia King Saud University Riyadh Saudi Arabia; 2 Department of Plant Protection, Ghazi University, Dera Ghazi Khan, Punjab, Pakistan Ghazi University Dera Ghazi Khan Pakistan; 3 Departamento de Entomologia e Acarologia, Escola Superior de Agricultura “Luiz de Queiroz”, Universidade de São Paulo, São Paulo, Brazil Universidade de São Paulo São Paulo Brazil

**Keywords:** *Asparagi*, *schizopus*, tactile setae, *taquarae*, *vermiculatus*

## Abstract

After a comprehensive taxonomic assessment of descriptions/ illustrations of all known (118) species of the spider mite genus *Schizotetranychus* Trägårdh (Acari: Prostigmata: Tetranychidae), five species groups are proposed based on the number of tactile setae on tibia II in female, i.e., *vermiculatus* with four setae (four spp.), *schizopus* with five setae (52 spp.), *spireafolia* with six setae (10 spp.), *asparagi* with seven setae (20 spp.), and *bambusae* with eight setae on tibia II (22 spp.). The species group *schizopus* is further divided into three species subgroups based on tactile setae on tibia I: *schizopus* with eight/ nine setae (21 spp.), *andropogoni* with seven setae (26 spp.), and *taquarae* with six tactile setae excluding the solenidion on tibia I (five spp.). Eight *Schizotetranychus* species were not assigned to any species group because of brief descriptions and/ or illustration and without information on the number of tactile setae on tibiae I and II. Moreover, two *Schizotetranychus* species, *S.gausus* Baker & Pritchard and *S.luculentus* Tseng that have six setae/ structures including a spinneret and a solenidion on the palp tarsus, are provisionally transferred to the genus *Stigmaeopsis* Banks. Finally, keys to species groups and subgroups of the world species of *Schizotetranychus* are provided.

## ﻿Introduction

The genus *Schizotetranychus* Trägårdh (Acari, Prostigmata, Tetranychidae) was erected by Trägårdh (1915) based on the shape of leg empodia, i.e., divided deeply into two claw-like structures and having ten pairs of dorsal hysterosomal setae. It is one of the largest genera of spider mites containing 118 species, widely distributed in the world (Migeon and Dorkeld 2006–2024). *Schizotetranychus* species are phytophagous on different plant species and some species are considered as pests of agricultural crops, i.e., *Schizotetranychusandropogoni* (Hirst) and *Schizotetranychusasparagi* (Oudemans, 1928) are widespread in United States and Europe causing serious infestations to pineapple plants (Jeppson et al. 1975; Hoy 2011).

*Schizotetranychus* species identity has been challenging due to the inadequate number of diagnostic characters, minute differences in male aedeagus morphology, and interspecific similarities in females of many species. Specimens of both sexes are usually required for accurate identification of *Schizotetranychus* species (Pritchard and Baker 1955; Meyer 1974, 1987; Jeppson et al. 1975; Flechtmann 2012). Pritchard and Baker (1955) and Meyer (1974, 1987) provided only diagnostic keys of *Schizotetranychus* species occurring in some regions. Lastly, Flechtmann (2012) arbitrarily organized the *Schizotetranychus* species into 17 groups only based on female morphology; however, the identification characters used are confusing. Additionally, a diagnostic key to world *Schizotetranychus* species is not available. The present study aimed to classify the species of the genus *Schizotetranychus* into species group and subgroups based on consistent diagnostic morphological characters, and to develop diagnostic keys to species groups and subgroups of world species of the genus *Schizotetranychus*.

## ﻿Materials and methods

The taxonomic literature of all known *Schizotetranychus* species were critically studied, and the diagnostic characters were compared. The generic characters of *Schizotetranychus* and *Stigmaeopsis* were carefully analyzed for possible new combinations. The strength of each morphological character was evaluated for its suitability at the specific level. The consistency of tibia I and II setal counts were carefully evaluated for the construction of species and sub-species group. The key to species of the genus *Schizotetranychus* is provided based on persistent and fixed characteristics.

## ﻿Results and discussion

**Subfamily Tetranychinae Berlese**

**Tribe Tetranychini Reck**

### ﻿Genus Schizotetranychus

Taxon classificationAnimaliaTrombidiformesTetranychidae

Trägårdh

ACE9D756-FA61-5466-9DD9-F1CDC4E7C863

#### Type species.

*Tetranychusschizopus* Zacher, 1913.

#### Diagnosis.

Dorsal hysterosoma with ten pairs of setae (*c_1-3_*, *d_1-2_*, *e_1-2_*, *f_1-2_*, and *h_1_*), setae *h_2_* and *h_3_* present on ventral opisthosoma, empodial claw divided deeply into two claw-like structures, palp tarsus with seven structures/ setae (one spinneret, two eupathidia, one solenidion, three setiform setae); dorsal hysterosoma medially usually with transverse striations, but may be longitudinal or irregular between *d_1_* and *e_1_*; two sets of duplex setae on tarsus I present distally, nearly adjacent to each other.

##### ﻿Background and taxonomic review of the genus *Schizotetranychus*

The genus *Schizotetranychus* was erected by Trägårdh (1915) based on *Tetranychusschizopus* Zacher, 1913 and distinguished from the genus *Tetranychus* by having the leg empodia divided deeply into two claw-like structures. Two years later, Banks (1917) erected the genus *Stigmaeopsis* and designated *St. celarius* Banks its type species. Banks (1917) described the genus *Stigmaeopsis* very briefly and did not provide any diagnostic characters which could separate it from the closely related genus *Schizotetranychus*. Later, McGregor (1950) synonymized the monospecific genus *Stigmaeopsis* with *Schizotetranychus*. This synonymy was accepted and followed by Pritchard and Baker (1955), Baker and Pritchard (1960), Tuttle and Baker (1968), Gutierrez (1968), Meyer (1974, 1987), Tuttle et al. (1976), Bolland et al. (1998), and Ehara (1999).

For the first time, Ehara (1999) introduced species groups in the genus *Schizotetranychus* by dividing the nine *Schizotetranychus* species reported from Japan into two species groups: *schizopus* (six species) with transverse striations in the anterior portion of dorsocentral area on dorsal opisthosoma and *celarius* (three species) with longitudinal striations in the anterior portion of dorsocentral area on dorsal opisthosoma.

Later, Saito et al. (2004) reinstated the genus *Stigmaeopsis* and distinguished it from *Schizotetranychus* and other genera of Tetranychidae by having six setae/ structures on the palp tarsus in the female (instead of seven); dorsal striations between *c_1_* and *d_1_*, clearly longitudinal, forming a trapezoidal shape instead of having mostly transverse or longitudinal irregular without forming a trapezoidal shape in *Schizotetranychus*. Also, the bases of setae *e_1_*, *d_1_*, and *c_1_* gradually become further apart than the bases of *f_1_* setae; if hypothetical lines connecting their bases are drawn, they form a V-shaped pattern vs these lines being almost parallel in *Schizotetranychus* and related genera, as described by Saito et al. (2004). Based on these characteristics, Flechtmann (2012) transferred two *Schizotetranychus* species, *S.malkovskii* Wainstein, 1956 and *S.meghalensis* (Gupta & Gupta, 1994) to *Stigmaeopsis*. Although *S.meghalensis* has transverse striations between setae *c_1_*, *d_1_*, and *e_1_*, and does not satisfy several characters of *Stigmaeopsis*, despite the fact that Saito et al. (2016, 2018, 2019) provisionally included this species in *Stigmaeopsis* because of its six setae/ structures on the palp tarsus.

##### ﻿Morphological diagnostic features previously used for grouping *Schizotetranychus* species

Flechtmann (2012) categorized world 106 *Schizotetranychus* species into 17 groups based on following female morphological characters: body length: width ratio, dorsal setal length, shape of peritremes, number of tactile setae on tibia I. As a result, numerous species groups in the genus *Schizotetranychus* based on variable morphological characters are causing confusion and misunderstanding in species identification.

1. Peritremes distally are variously developed in *Schizotetranychus* species, straight in most species, and either making a U-shape, ring, or looped distally others. Peritremes distally are anastomosing in two species *S.cajani* Gupta, 1996, *S.prosopis* Tuttle, Baker & Abbatiello, 1976. Flechtmann (2012) used this character to arbitrarily propose different groups for *Schizotetranychus* species. We consider the shape of the peritreme at species level a misleading character because it is variously developed distally even in different specimens of the same species, and described and illustrated differently for one species by various authors. Also, this character was already causing confusion while attempting to separate the species groups of *Schizotetranychus* created by Flechtmann (2012).
2. Mite body shape is either oval (longer than wide in most of species), or orbicular (as long as wide in few species), or elongate (more than 2× longer than width of body in few species). This character was used by Flechtmann (2012) to develop groups in *Schizotetranychus*. However, it caused confusion in the identification of those groups because some species lie on the borderline in length and width ratios. Also, the length and width ratios could be affected by the mounting of specimens on glass slides.
3. Dorsal and ventral idiosoma is entirely striated in almost all *Schizotetranychus* species, either widely or closely spaced, except *S.reticulatus* Baker & Pritchard, 1960 with reticulations on the propodosoma medially, and the hysterosoma is entirely striated and rugose. The dorsal hysterosoma between setae *c _1_* , *d _1_* , *e _1_* , and *f _1_* with transverse striations entirely in all *Schizotetranychus* species except six, namely *S.hidayahae* Yusof & Zhang, 2003, *S.baltazarae* Rimando, 1962, *S.spiculus* Baker & Pritchard, 1960, *S.brevisetosus* Ehara, 1989, *S.rhodanus* Baker & Pritchard, 1960, and *S.colocasiae* Ehara, 1988 (as in Ehara & Tho, 1988), in which the striations between setae *e _1_* and *d _1_* form a V-shaped pattern or is irregular.

Dorsal body setae are usually setiform in *Schizotetranychus* species. However, few species have awl-shaped dorsal setae with slightly expanded bases. Flechtmann (2012) used this character to develop species groups in *Schizotetranychus*. However, dorsal setae were not properly illustrated or described in detail for many *Schizotetranychus* species, so for those species it is very difficult to discern the exact shape (awl or setiform) of the dorsal setae. This character can be considered as supporting species level character.

##### ﻿Taxonomic notes about two *Schizotetranychus* species having six setae/ structures on the palp tarsus as in the genus *Stigmaeopsis*

As mentioned earlier, the genus *Stigmaeopsis* differs from *Schizotetranychus* by having six setae/ structures on the palp tarsus in females instead of seven; dorsal striations lie between *c_1_* and *d_1_* are clearly longitudinal and forming a trapezoidal shape instead of being mostly transverse or irregularly longitudinal between setae *d_1_* and *e_1_* in six *Schizotetranychus* species, namely, *S.hidayahae*, *S.baltazarae*, *S.spiculus*, *S.brevisetosus*, *S.rhodanus* and *S.colocasiae* without forming a trapezoidal shape. Also, the bases of setae *e_1_*, *d_1_*, and *c_1_* gradually widen further apart than the bases of *f_1_* setae if hypothetical lines connecting their bases are drawn. They form a V-shaped pattern vs almost parallel lines are in *Schizotetranychus* and related genera, as described by Saito et al. (2004).

In the present study, it was found that two *Schizotetranychus* species, *S.gausus* Baker & Pritchard, 1960 and *S.luculentus* (Tseng, 1990) have six setae/ structures including spinneret and solenidion on palp tarsus. The original description of these species lacking information of palp setae. So, relying on the original illustrations, these two *Schizotetranychus* species having six setae on palp tarsus are provisionally transferred to *Stigmaeopsis*. Also, dorsum is entirely reticulated in *S.luculentus* (Tseng, 1990). However, dorsum with transverse striations between setae *c_1_*, *d_1_* and irregular longitudinal between setae *e_1_* and *d_1_* in *S.gausus*. Moreover, 16 known species of *Stigmaeopsis* have five tactile setae on tibia II except *S.gausus* having seven setae on tibia II.

Furthermore, bases of length dorsal setae *c_1_*, *d_1_* which is ~ 2× more widely spaced to the bases of *e_1_* and *f_1_* (bases of *c_1_*, *d_1_*, *e_1_*, *f_1_* forming a V-shaped pattern) in *S.* Attiah, 1967 as in all known 16 *Stigmaeopsis* species. Few other *Schizotetranychus* species have a similar pattern of dorsal setal bases. So, the supporting diagnostic character that hypothetical lines connecting the bases of setae *c_1_*, *d_1_*, *e_1_*, and *f_1_* forming a V-shaped taken by Saito et al. (2004) for *Stigmaeopsis* while reinstating this genus to separate it from *Schizotetranychus* becomes impractical.

Hence it is understood from the above discussion that genus *Stigmaeopsis* is different from *Schizotetranychus* by only one character, the presence of six setae/ structures on palp tarsus vs seven in *Schizotetranychus*. All other supporting characters (longitudinal striations between setae *c_1_* and *d_1_*, bases of setae *c_1_*, *d_1_*, *e_1_*, and *f_1_* forming a V-shaped pattern) of *Stigmaeopsis* taken by Saito et al. (2004, 2018) as a generic character could be considered as species level characters.

##### ﻿Species groups and subgroups of *Schizotetranychus* developed in the current study

In the present research, after comprehensive taxonomic assessment of descriptions and illustrations of all known (116) species of the genus *Schizotetranychus*, species grouping in this genus is reconsidered based on females using only the number of tactile setae on tibia II and species subgroups based on only the number of tactile setae on tibia I. The number of tactile setae on tibia II is found to be a consistent diagnostic character in *Schizotetranychus* species and described in 110 *Schizotetranychus* species, even those which were very briefly described. Flechtmann (2012) used tactile setae on tibia I to separate some *Schizotetranychus* groups. Pritchard and Baker (1955) and Mushtaq et al. (2021) also used tactile setae on tibia I to develop species groups in the genus *Oligonychus*. Based on tibial setal counts, species groups of *Schizotetranychus* can easily be recognized.

In the present study, the genus *Schizotetranychus* can be divided into five species groups based on the number of tactile setae on tibia II in the female: *schizopus* group (52 spp.) with five setae, *asparagi* group with seven setae (20 spp.), *bambusae* group with eight setae (22 spp.), *spireafolia* group with six setae (10 spp.) and *vermiculatus* group with four setae on tibia II (four spp.). Also, keys to the world *Schizotetranychus* species, species groups, and subgroups are developed for the first time. Eight *Schizotetranychus* species were not assigned any species group because these have been described and illustrated very briefly without information on the number of setae on tibia I and II.

**1. Species group *schizopus***

**Diagnosis. Female**: Tibia II with five setae (52 species).

**Exemplar species.***Schizotetranychusschizopus* (Zacher, 1913)

Species group *schizopus* is further divided into three species subgroups based on number of tactile setae excluding solenidion on tibia I.

**i) Species subgroup *schizopus***

**Diagnosis. Female.** Tibia I with eight/ nine setae (21 species).

**Exemplar species.** *Schizotetranychusschizopus*

**ii) Species subgroup *andropogoni***

**Diagnosis. Female.** Tibia I with seven setae (26 species).

**Exemplar species.***Schizotetranychusandropogoni* (Hirst, 1926)

**iii) Species subgroup *taquarae***

**Diagnosis. Female.** Tibia I with six setae (5 species).

**Exemplar species.***Schizotetranychustaquarae* Paschoal, 1971

**2. Species group *asparagi***

**Diagnosis. Female.** Tibia II with seven setae (20 spp.)

**Exemplar species.***Schizotetranychusasparagi* (Oudemans, 1928)

**3. Species group *bambusae***

**Diagnosis. Female.** Tibia II with eight setae (22 spp.).

**Exemplar species.***Schizotetranychusbambusae* Reck, 1941

**4. Species group *spireafolia***

**Diagnosis. Female.** Tibia II with six setae (10 spp.).

**Exemplar species.***Schizotetranychusspireafolia* Garman, 1940

**5. Species group *vermiculatus***

**Diagnosis. Female.** Tibia II with four setae (04 spp.).

**Exemplar species.***Schizotetranychusvermiculatus* Ehara & Wongsiri, 1975

##### ﻿Ungrouped species

The following eight species were not assigned any species group because these have been described and illustrated very briefly without information of number of tactile setae on tibiae I and II. *Schizotetranychussetariae* Meyer, 1987 was not assigned to any species group/ subgroup because it was only described/ known from the male.

1. *S.graminicola* Goux, 1949
2. *S.glabrisetus* (Ugarov & Nikolskii, 1937)
3. *S.tuberculatus* (Ugarov & Nikolski, 1937)
4. *S.guatemalae-novae* (Stoll, 1886)
5. *S.hindustanicus* (Hirst, 1924)
6. *S.mustafaii* Mustafa & Chaudri, 1972 (as in Mustafa et al. 1972)
7. *S.oudemansi* Reck, 1948
8. *S.setariae* Meyer, 1987

Moreover, two *Schizotetranychus* species, *S.gausus* and *S.luculentus*, that have six setae/ structures including spinneret and a solenidion on the palp tarsus based on original illustrations, are provisionally transferred to *Stigmaeopsis*. Further studies are required to confirm the clear taxonomic status of these two species.

### ﻿1. *Schizopus* species group (52 species)

#### ﻿I- *Schizopus* species subgroup (21 species)

(Female: tibia II with 5 setae, tibia I with 8 or 9 tactile setae excluding solenidion)

### ﻿Key to species of *schizopus* species subgroup of *schizopus* species group

**Table:** 

1	Propodosoma dorsomedially with reticulate pattern	***S.reticulatus* Baker & Pritchard, 1960**
–	Propodosoma dorsomedially striated	**2**
2	Peritremes distally curved U- or ring-shaped/ looped	**3**
–	Peritremes distally straight or slightly hooked	**7**
3	Tibia I with 9 tactile setae and 1 sensory seta	*S.australis* Gutierrez, 1968
–	Tibia I with 8 tactile setae and 1 sensory seta	**4**
4	Dorso-opisthosomal setae *c_1_*, *d_1_*, *e_1_* at least reaching 1/2 to 2/3 of the setae next in line	***S.pennamontanus* Meyer, 1987**
–	Dorso-opisthosomal setae *c_1_*, *d_1_*, *e_1_* almost reaching or crossing the setae next in line	**5**
5	Male aedeagus upturned part not sigmoid, distal part not projecting posteriorly	***S.russeus* Davis, 1969**
–	Male aedeagus upturned part sigmoid, distal part projecting posteriorly	**6**
6	In male: tarsus I with 3 and tibia I with 1 spindle-shaped setae; in female: setae *c_1_*, *d_1_*, *e_1_* at least reaching the setae next in line	***S.eremophilus* McGregor, 1950**
–	In male: tarsus I and tibia I without spindle-shaped setae; in female: setae *c_1_*, *d_1_*, *e_1_* crossing the setae next in line	***S.elymus* McGregor, 1950**
7	Tibia I with 9 tactile setae and 1 solenidion	**8**
–	Tibia I with 8 tactile setae and 1 solenidion	**10**
8	Male: aedeagus upturned part almost sigmoid, without anterior projection, tibia I with 7 tactile setae and 2 solenidia	***S.mansoni* Gupta, 1980**
–	Male: aedeagus upturned part, not sigmoid, with anterior projection, tibia I with 9 tactile setae and 3 or 4 solenidia	**9**
9	Male: tibia I with 9 tactile setae and 3 solenidia, tibia II with 5 tactile setae	***S.schizopus* (Zacher, 1913)**
–	Male: tibia I with 9 tactile setae and 4 solenidia, tibia II with 6 tactile setae	***S.lechrius* Rimando, 1962**
10	Female: genu III with 4 tactile setae	**11**
–	Female: genu III with 3 tactile setae	**14**
11	Female: setae *c_1_*, *d_1_*, *e_1_* far behind the bases of setae next in line	***S.agropyron* Tuttle & Baker, 1976**
–	Female: setae *c_1_*, *d_1_*, *e_1_* crossing the bases of setae next in line	**12**
12	Male: aedeagus upturned part at right or acute angle to the shaft, does not project posteriorly	***S.nesbitti* Meyer, 1965**
–	Male: aedeagus upturned part making obtuse angle to the shaft, projecting posteriorly	**13**
13	Male: aedeagus not sigmoid, upturned part gradually narrowing toward distal end, 1.5× longer than max. width of shaft	***S.cynodonis* McGregor, 1950**
–	Male: aedeagus almost sigmoid, upturned part abruptly narrowing toward distal end, less than max. width of shaft	***S.paezi* Alvarado & Freitez, 1976**
14	Female: femur I with 7 or 8 setae	**15**
–	Female: femur I with 9 or 10 setae	**17**
15	Female: genu IV with 2 setae	***S.echinulatus* Mitrofanov, 1978**
–	Female: genu IV with 3 setae	**16**
16	Dorso-central hysterosomal setae shorter than distance/interval to base of seta immediately behind	***S.saba-sulchani* Reck, 1956**
–	Dorso-central hysterosomal setae as long as or longer than distance/interval to base of seta immediately behind	***S.yoshimekii* Ehara & Wongsiri, 1975**
17	Femur I with 9 setae	**18**
–	Femur I with 10 setae	**19**
18	Female: tibia III with 5 setae. Male: aedeagus upturned part with knob, prominent neck, and anterior projection	***S.hilariae* Tuttle & Baker, 1968**
–	Female: tibia III with 5 setae. Male: aedeagus upturned part without knob, neck, or anterior projection	***S.tuttleii* Zaher, Gomaa & El-Enany, 1982**
19	Female: femur III with 2 setae, peritremes L-shaped distally. Male: aedeagus upturned part length of shaft less than max. width of shaft	***S.kochummeni* Ehara, 1988 (as in Ehara and Tho 1988)**
–	Female: femur III with 3 setae, peritremes straight distally. Male: aedeagus upturned part of shaft length > 2× the max. width of shaft	**20**
20	Female: femur IV with 2 setae. Male: aedeagus upturned part almost at right angle to the shaft, not projecting posteriorly	***S.tbilisiensis* Reck, 1959**
–	Female: femur IV with 3 setae. Male: aedeagus upturned part making obtuse angle (120°) to the shaft, projecting posteriorly	***S.tumidus* Wang, 1981**

#### ﻿II- *andropogoni* species subgroup (26 species)

(Female: tibia II with 5 setae, tibia I with 7 tactile setae excluding solenidion)

### ﻿Key to species of *andropogoni* species subgroup

**Table:** 

1	Peritremes distally hooked, branched, or looped	**10**
–	Peritremes distally simple (bulb-like)	**2**
–	Peritremes distally anastomosing	***S.cajani* Gupta, 1996**
2	Dorsal setae comparatively shorter in length, setae *c_1_* reaching at least 2/3 the distance *c_1_*-*d_1_*	**3**
–	Dorsal setae comparatively long, setae *c_1_* reaching/ crossing the bases of *d_1_*, almost equal to/ longer than the distance *c_1_*-*d_1_*	**7**
3	Idiosoma elongate, ratio of body length (not including rostrum): width > 2	**4**
–	Idiosoma oval/ orbicular, ratio of body length (not including rostrum): width < 2	**5**
4	Tarsus I with 3 tactile setae and 1 solenidion proximal to proximal duplex setae	***S.lycurus* Tuttle & Baker, 1964**
–	Tarsus I with 1 tactile seta and 1 solenidion proximal to proximal duplex setae	***S.boutelouae* Tuttle & Baker, 1968**
5	Femur I with 7 setae, area anterior to genital flap with transverse striations, tarsus I with 2 tactile setae and 7 solenidion proximal to proximal duplex setae	***S.celtidis* Tuttle & Baker, 1968**
–	Femur I with 9 setae, area anterior to genital flap with longitudinal striations, tarsus I with 3 tactile setae and 1 solenidion proximal to proximal duplex setae	**6**
6	Genu III with 2 setae, genu IV with 1 seta, area on hysterosoma between *d_1_* and *e_1_* with longitudinal striations	***S.hidayahae* Yusof & Zhang, 2003**
–	Genua III and IV each with 3 setae, hysterosoma dorsomedially with transverse striations entirely	***S.montanae* Tuttle & Baker, 1968**
7	Setae *c_1_* very long, crossing the bases of *d_1_*, reaching to the bases of *e_1_*	***S.longirostris* Feres & Flechtmann, 1995**
–	Setae *c_1_* reaching at least to the bases of *d_1_*, almost equal to the distance *c_1_*-*d_1_*	**8**
8	Area anterior to genital flap with longitudinal irregular striations	***S.paraelymus* Feres & Flechtmann, 1995**
–	Area anterior to genital flap with transverse striations	**9**
9	In female: seta *sc_1_* much longer than *sc_2_*, setae *d_1_* and *e_1_* not reaching to the bases of setae next in line. Male: tarsus I with 3 solenidia and 1 tactile seta proximal to proximal duplex setae, aedeagus upturned part sigmoid without anterior projection	***S.camur* Pritchard & Baker, 1955**
–	In female: setae *sc_1_* and *sc_2_* almost subequal, setae *d_1_* and *e_1_* reaching to the bases of setae next in line. Male: tarsus I with 1 solenidion and 1 tactile seta proximal to proximal duplex setae, aedeagus upturned part with anterior projection	***S.andropogoni* (Hirst, 1926)**
10	Dorso-central setae *c_1_*, *d_1_*, and *e_1_* reaching or crossing the bases of next setae in line	**11**
–	Dorso-central setae *c_1_*, *d_1_*, and *e_1_* not reaching behind the bases of next setae in line	**16**
11	Female: tarsus I with 2 or 4 setae and 1 solenidion proximal to proximal duplex setae	**12**
–	Female: tarsus I with 1 seta and 1 solenidion proximal to proximal duplex setae	**14**
12	Female: tarsus I with 4 setae and 1 solenidion proximal to proximal duplex setae	***S.filifolius* Meyer, 1974**
–	Female: tarsus I with 2 setae and 1 solenidion proximal to proximal duplex setae	**13**
13	Female: All hysterosomal setae longer than longitudinal interval between their bases. Male: tarsus I with 2 setae and 2 solenidia proximal to proximal duplex setae, upturned part of aedeagus making almost right angle with shaft	***S.sacharum* Flechtmann & Baker, 1975**
–	Female: most of hysterosomal setae approximately as long as the longitudinal interval between their bases. Male: tarsus I with 3 setae and 2 solenidia proximal to proximal duplex setae, upturned part of aedeagus making acute angle with shaft	***S.youngi* Tseng, 1975**
14	Female: hysterosomal setae especially *c_1_*, *d_1_*, *e_1_* barely reaching the bases of next setae in line. Male: tarsus I with 1 solenidion and 1 tactile seta proximal to proximal duplex setae	***S.krungthepensis* Auger & Naing, 2014 (as in Naing et al. 2014)**
–	Female: hysterosomal setae especially *c_1_*, *d_1_*, *e_1_* longer than distance to bases of next setae in line. Male: tarsus I with 2 or 3 solenidia and 1 tactile seta proximal to proximal duplex setae	**15**
15	Male: tibia I with 7 tactile setae and 3 solenidia, tibia II with 5 tactile setae and 1 solenidion, tarsus I with 1 tactile seta and 3 solenidia proximal to proximal duplex setae	***S.arcuatus* Meyer, 1974**
–	Male: tibia I with 7 tactile setae and 4 solenidia, tibia II with 5 tactile setae only without solenidion, tarsus I with 1 tactile seta and 2 solenidia proximal to proximal duplex setae	***S.rhynosperus* Flechtmann & Baker, 1970**
16	Female: Dorsal hysterosoma medially with transverse striations except area between setae *c_1_*, *d_1_*, and *e_1_* forming V-shaped or longitudinal pattern	**17**
–	Female: Dorsal hysterosoma medially with transverse striations entirely	**19**
17	Female: Stylophore anteriorly emarginate, with notch	**18**
–	Female: Stylophore anteriorly without notch	***S.sacrales* Baker & Pritchard, 1960**
18	Female: Tarsus I with 2 solenidia and two tactile setae proximal to proximal duplex setae; striations in between setae *c_1_* to *d_1_* longitudinal	***S.baltazarae* Rimando, 1962**
–	Female: Tarsus I with 1 solenidion and 2 tactile setae proximal to proximal duplex setae; striations in between setae *c_1_* to *d_1_* transverse	***S.spiculus* Baker & Pritchard, 1960**
19	Peritremes looped (making a loop) distally	***S.nugax* Pritchard & Baker, 1955**
–	Peritremes hooked or making L-shape distally	**20**
20	Idiosoma elongate, ratio of body length (not including rostrum): width > 2	**23**
–	Idiosoma oval/ orbicular, ratio of body length (not including rostrum): width < 2	**21**
21	Female: Dorsal striations smooth without lobes. Male: aedeagus upturned part making right angle with the shaft	***S.sagatus* Davis, 1969**
–	Female: Dorsal striations with lobes. Male: aedeagus upturned part making obtuse angle with the shaft	**22**
22	Femur I with 7 setae, stylophore notched anteriorly, striations in pregenital area making strongly arched	***S.denmarki* Baker & Tuttle, 1994**
–	Femur I with 9 setae, stylophore rounded anteriorly, striations in pregenital area transverse	***S.pseudolycurus* Ochoa, Gray & von Lindeman, 1990**
23	Tarsus I with 4 tactile setae proximal to proximal duplex	***S.fluvialis* McGregor, 1928**
–	Tarsus I with 3 tactile setae proximal to proximal duplex	**24**
24	Striations in pregenital area almost transverse slightly curved	***S.freitezi* Ochoa, Gray & von Lindeman, 1990**
–	Striations in pregenital area longitudinal irregular	***S.oryzae* Rossi de Simons, 1966**

#### ﻿III- *taquarae* species subgroup (05 species)

(Female: tibia II with 5 setae, tibia I with 6 tactile setae excluding solenidion)

### ﻿Key to species of *taquarae* species subgroup

**Table:** 

1	Dorsal setae very short, far behind the bases of next setae in line	**2**
–	Dorsal setae long, at least reaching or crossing the bases of next setae in line	**3**
2	Female: peritremes hooked distally, dorsal striations without lobes, striations on pregenital area transverse; femur IV with 3 setae. Male: aedeagus upturned part sigmoid without anterior projection	***S.tegophallos* Flechtmann & Peralta-Alba, 2012**
–	Female: peritremes simple, without hook distally, dorsal striations with lobes, striations on pregenital area longitudinal; femur IV with 2 setae. Male: aedeagus upturned part not sigmoid with anterior projection	***S.umtaliensis* Meyer, 1974**
3	Female: tarsus I with 4 tactile setae and a solenidion proximal to proximal duplex setae. Male: aedeagus not sigmoid, shaft almost straight, narrowing toward distal end	***S.triquetrus* Meyer, 1987**
–	Female: tarsus I with 2 or 3 tactile setae and a solenidion proximal to proximal duplex setae. Male: aedeagus almost sigmoid, upturned	**4**
4	Dorsal striations with lobes, tarsus I with 2 tactile setae and a solenidion proximal to proximal duplex setae	***S.taquarae* Paschoal, 1971**
–	Dorsal striations without lobes, tarsus I with 3 tactile setae proximal to proximal duplex setae	***S.papillatus* Flechtmann, 1995**

### ﻿2. *asparagi* species group (20 species)

(Female: tibia II with 7 setae)

#### Key to species (20) of *asparagi* species group

**Table:** 

1	Tibia I with 12 setae including solenidia	**2**
–	Tibia I with 8–10 setae and 1 solenidion	**3**
2	Female: femur IV with 4 setae, genua III and IV with 3 setae. Male: aedeagus downturned, with only posterior projection	***S.emeiensis* Wang, 1983**
–	Female: femur IV with 4 setae, genua III and IV with 3 setae. Male: aedeagus upturned, with anterior and posterior projections	***S.kreiteri* Flechtmann, 1999 (as in Flechtmann et al. 1999)**
3	Tibia I with 7 or 8 setae and a solenidion	**4**
–	Tibia I with 9 setae and a solenidion	**7**
4	Tibia I with 7 tactile setae and a solenidion	***S.lanyuensis* Tseng, 1975**
–	Tibia I with 8 tactile setae and a solenidion	**5**
5	Dorsal hysterosoma medially with transverse striations entirely, dorsal setae, especially *c_1_*, *d_1_*, *e_1_*, longer than interval between their bases	***S.miyatahus* (Meyer, 1974)**
–	Dorsal hysterosoma medially between setae *d_1_* and *e_1_* with longitudinal irregular striations, dorsal setae shorter than interval between their bases	**6**
6	Female: Dorsal setae serrated, tarsus I with 5 tactile setae and 1 solenidion proximal to proximal duplex setae. Male: aedeagus upturned with small anterior projection and long posterior projection	***S.brevisetosus* Ehara, 1989**
–	Female: Dorsal setae nude, tarsus I with four tactile setae and a solenidion proximal to proximal duplex setae. Male: aedeagus almost straight without anterior projection	***S.rhodanus* Baker & Pritchard, 1960**
7	Femur I with 10 setae	**8**
–	Femur I with 8 or 9 setae	**12**
8	Tibia IV with 6 setae	**9**
–	Tibia IV with 7 setae	**10**
9	Peritremes hooked distally	***S.lushanensis* Wang, 1994**
–	Peritremes simple/ straight distally	***S.zhangi* Wang & Cui, 1992**
10	Femur II with 6 setae, femur IV with 4 setae	***S.kaspari* Manson, 1967**
–	Femur II with 7 setae, femur IV with 2 or 3 setae	**11**
11	Femur III with 4 setae, femur IV with 3 setae	***S.tuminicus* Ma & Yuan, 1982**
–	Femur III with 3 setae, femur IV with 2 setae	***S.halimodendri* Wainstein, 1958**
12	Femur I with 8 setae	***S.sayedi* Attiah, 1967**
–	Femur I with 9 setae	**13**
13	Tibia III with 5 setae	**14**
–	Tibia III with 6 setae	**15**
14	Female: Tibia IV with 5 setae, femur IV with 4 setae. Male: aedeagus distal part downturned, with small posterior projection	***S.asparagi* (Oudemans, 1928)**
–	Female: Tibia IV with 6 setae, femur IV with 3 setae. Male: aedeagus distal part upturned, with large posterior projection	***S.tephrosiae* Gutierrez, 1968**
15	Genu IV with 3 setae	***S.lespedezae* Beglyarov & Mitrofanov, 1973**
–	Genu IV with 4 setae	**16**
16	Striations on dorsal hysterosoma medially between setae *e_1_* forming V-shaped or irregular longitudinal patterns	**17**
–	Dorsal hysterosoma medially with entirely transverse striations	**18**
17	Female. Dorsal setae *c_1_*, *d_1_*, and *e_1_* just reaching the bases of next consecutive setae, peritremes slightly hooked distally. Male. Aedeagus with very minute anterior projection, aedeagal knob making acute angle with shaft	***S.colocasiae* Ehara, 1988 (as in Ehara and Tho 1988)**
–	Female. Dorsal setae *c_1_*, *d_1_* and *e_1_* crossing the bases of next consecutive setae, peritremes almost straight distally. Male. Aedeagus with very prominent anterior projection, aedeagal knob making obtuse angle with shaft	***S.malodhensis* Sadana et al., 1985**
18	Setae *c_1_* and *d_1_* reaching maximum up to 2/3 distance to setae next in line	***S.protectus* Meyer, 1965**
–	Setae *c_1_* and *d_1_* as long as or crossing the bases of setae next in line	**19**
19	Male: Eupathidium on palp tarsus absent, aedeagus knob of upturned part parallel with the shaft	***S.malayanus* Ehara, 1988 (as in Ehara and Tho 1988)**
–	Male: Eupathidium on palp tarsus present, aedeagus knob of upturned part making obtuse angle with the shaft	***S.bhandhufalcki* Ehara & Wongsiri, 1975**

### ﻿3. *bambusae* species group (22 species)

(Female: Tibia II with 8 setae)

### ﻿Key to species of *bambusae* species group

**Table:** 

1	Tibia I with 10 or 11 setae including solenidion	**2**
–	Tibia I with 9 setae including solenidion	***S.indicus* Gupta & Gupta, 1994**
2	Peritremes distally hooked, U/L-shaped	**3**
–	Preitremes almost straight, slightly expanded distally, not hooked/ U/L-shaped distally	**11**
3	Genua III and IV each with 3 setae	**4**
–	Genua III and IV each with 4 setae	**7**
4	Female: Dorsal setae comparatively short, far behind the bases of next setae in line. Male: aedeagus distal part upturned with anterior projection	***S.gilvus* Ehara & Ohashi, 2005**
–	Female: Dorsal setae long, crossing the bases of next setae in line. Male: aedeagus distal part straight undulating or downturned without anterior projection	**5**
5	Female: setae *c_1_* just crossing setae *d_1_*, far behind the bases of setae *e_1_*, tarsus I with 2 or 3 tactile setae and a solenidion proximal to proximal duplex setae	***S.minutus* Wang, 1985 (as mentioned in Wang et al. 1985)**
–	Female: setae *c_1_* reaching the bases of setae *e_1_*, tarsus I with 4 or 5 tactile setae and 1 solenidion proximal to proximal duplex setae	**6**
6	Female: pregenital area with longitudinal striations, tarsus I with 4 tactile setae and 1 solenidion proximal to proximal duplex setae. Male: aedeagus distal part almost straight, undulating, slightly turning up	***S.gahniae* Davis, 1969**
–	Female: pregenital area with transverse striations, tarsus I with 5 tactile setae and 1 solenidion proximal to proximal duplex setae. Male: aedeagus distal part almost straight, downturned	***S.cercidiphylli* Ehara, 1973**
7	Femur IV with 3 setae	**8**
–	Femur IV with 4 setae	**9**
8	Femur II with 6 setae, peritremes V-shaped distally	***S.imperatae* Wang, 1983**
–	Femur II with 7 setae, peritremes L-shaped distally, slightly hooked	***S.textor* Wainstein, 1954**
9	Female: dorsal setae especially *c_1_*, *d_1_*, *e_1_* almost reaching to the bases of setae next in line. Male: aedeagus distal part downturned and sigmoid	***S.fauveli* Gutierrez, 1978**
–	Female: dorsal setae especially *c_1_*, *d_1_*, *e_1_* well crossing to the bases of setae next in line. Male: aedeagus distal part not sigmoid, almost straight/ undulating	**10**
10	Male: aedeagus distal part very long needle-like undulating	***S.alni* Beglyarov & Mitrofanov, 1973**
–	Male: aedeagus distal part downturned slightly gradually narrowing toward distal end, not needle-like	***S.zhongdianensis* Wang & Cui, 1992**
11	Tibia I with 10 tactile setae and 1 solenidion	**12**
–	Tibia I with 9 tactile setae and 1 solenidion	**13**
12	Female: femur I with 9 setae, femur II with 6 setae genu III with 3 setae. Male: aedeagus distal part straight undulating	***S.garmani* Pritchard & Baker, 1955**
–	Female: femur I with 10 setae, femur II with 7 setae, genu III with 4 setae. Male: aedeagus distal part slightly upturned, dorsally making slight knob, then bent down distally	***S.levinensis* Manson, 1967**
13	Male: aedeagus distal part upturned	**14**
–	Male: aedeagus distal part, straight or downturned	**18**
14	Male: aedeagus distal upturned part with prominent anterior projection and long posterior projection	**15**
–	Male: aedeagus distal upturned part without anterior projection	**16**
15	Eupathidium on male palp tarsus long, almost as long as eupathidia aedeagus posterior projection of upturned part is 4× longer than width of aedeagus neck and making prominent angle with neck	***S.beckeri* Wainstein, 1958**
–	Eupathidium on male palp tarsus minute, eupathidia 3× longer than, aedeagus posterior projection of upturned part is 2–3× longer than width of neck and not making angle with neck	***S.brachypodii* Livshits & Mitrofanov, 1968**
16	Male: aedeagus distal part turn dorso-caudally, almost sigmoid in shape	***S.ibericus* Reck, 1947**
–	Male: aedeagus distal part turn dorsally, not sigmoid in shape	**17**
17	Female: pregenital area with transverse striations, tarsus I with 4 tactile setae and a solenidion, tarsus II with 4 setae and a solenidion proximal to proximal duplex setae. Male: aedeagus upturned part greatly narrowing, needle-like	***S.floresi* Rimando, 1962**
–	Female: pregenital area with longitudinal striations, tarsus I with 5 tactile setae and 1 solenidion, tarsus II with 3 setae and 1 solenidion proximal to proximal duplex setae. Male: aedeagus upturned part blunt distally, not narrowing	***S.bambusae* Reck, 1941**
18	Male: aedeagus distal part straight, undulating	***S.jachontovi* Reck, 1953**
–	Male: aedeagus distal part down turned	**19**
19.	Female: femur IV with 3 setae. Male: aedeagus distal part slightly downturned without anterior projection	**20**
–	Female: femur IV with 4 setae. Male: aedeagus distal downturned part with distal knob, neck, and anterior projection	**21**
20	Female: tarsus I with 19 and tarsus II with 16 setae. Male: eupathidium su on palp tarsus long, longer than eupathidia	***S.smirnovi* Wainstein, 1954**
–	Female: tarsus I with 18 and tarsus II with 15 setae. Male: eupathidium su on palp tarsus almost half in length than eupathidia	***S.iraniensis* Mahdavi & Asadi, 2015**
21	Male: aedeagus anterior and posterior projections almost equal, knob forming obtuse angle with the shaft	***S.chiangmaiensis* Ehara & Wongsiri, 1975**
–	Male: aedeagus posterior projections much longer (2–3×) than anterior projection, knob forming acute angle with the shaft	***S.euphorbiae* Livshitz & Mitrofanov, 1968**

### ﻿4. *vermiculatus* species group (4 species)

(Female: Tibia II with 4 setae)

### ﻿Key to species of *vermiculatus* species group

**Table:** 

1	Tibia I with 7 setae and a solenidion, tibiae III and IV each with 4 setae	***S.vermiculatus* Ehara & Wongsiri, 1975**
–	Tibia I with 6 setae and a solenidion, tibiae III and IV each with 3 setae	**2**
2	Genua III and IV each with 3 setae	***S.approximatus* Ehara, 1988**
–	Genua III and IV each with 2 setae	**3**
3	Dorsocentral area between setae *c_1_*, *d_1_*, *e_1_*, and *f_1_* smooth, without striations	***S.laevidorsatus* Ehara, 1988**
–	Dorsocentral area between setae *c_1_*, *d_1_*, *e_1_*, and *f_1_* with transverse striations	***S.saitoi* Ehara, 1988**

### ﻿5. *spireafolia* species group (10 species)

(Female: Tibia II with 6 setae)

### ﻿Key to species of *spireafolia* species group

**Table:** 

1	Tibia I with 6 tactile setae and a solenidion	***S.prosopis* Tuttle, Baker & Abbatiello, 1976**
–	Tibia I with 7 tactile setae and a solenidion	**2**
–	Tibia I with 8 or 9 tactile setae and a solenidion	**4**
2	Dorsal setae very long, c*_1_* crossing the bases of *d_1_* reaching up to the bases of *e_1_*, setae *d_1_* reaching up to the bases of *f_1_*	***S.parasemus* Pritchard & Baker, 1955**
–	Dorsal setae, short, setae *c_1_* and *d_1_* and *e_1_* almost reaching up the bases of setae next in line or just crossing the bases of setae next in line	**3**
3	Female: Tarsus I with 2 setae and 1 solenidion proximal to proximal duplex setae, tarsus II with 1 tactile seta and 1 solenidion proximal to duplex setae. Male aedeagus upturned distal part shorter (less than half) the length of shaft	***S.recki* Ehara, 1957**
–	Female: Tarsus I with 5 tactile setae proximal to duplex setae, Tarsus II with 4 tactile setae and 1 solenidion proximal to duplex setae. Male aedeagus upturned distal part as long as the length of shaft	***S.undulatus* (Beer & Lang, 1958)**
4	Tibia I with 9 tactile setae and a solenidion	***S.ugarovi* Wainstein, 1960**
–	Tibia I with 8 tactile setae and a solenidion	**5**
5	Peritremes hooked distally. Male aedeagus upturned part with neck and anterior projection	***S.shii* Ehara, 1965**
–	Peritremes straight distally. Male aedeagus without anterior projection	**6**
6	Dorsal hysterosomal setae (most of them) awl-shaped, acutely tapering from the widened proximal (basal) portion	***S.spireafolia* Garman, 1940**
–	Dorsal hysterosomal setae setose	**7**
7	Tibiae III and IV each with 6 setae	***S.dalbergiae* Meyer, 1974**
–	Tibiae III and IV each with 5 setae	**8**
8	Femur II, III, and IV with 8, 4, and 2 setae, respectively. Male aedeagus upturned part as long as the length of shaft	***S.elongatus* Wang & Cui, 1991**
–	Femur II, III, and IV with 6, 3, and 3 setae, respectively. Male aedeagus upturned part very minute as compared to the length of shaft	***S.avetjanae* Bagdasarian, 1954**

## Supplementary Material

XML Treatment for Schizotetranychus

## ﻿Additional information

### Author ORCIDs

Muhammad Kamran https://orcid.org/0000-0001-6084-203X

Fahad Jaber Alatawi https://orcid.org/0000-0002-6824-2650

### Data availability

All of the data that support the findings of this study are available in the main text.
